# Prognostic factors for outcomes after whole-brain irradiation of brain metastases from relatively radioresistant tumors: a retrospective analysis

**DOI:** 10.1186/1471-2407-10-582

**Published:** 2010-10-26

**Authors:** Thekla Meyners, Christine Heisterkamp, Jan-Dirk Kueter, Theo Veninga, Lukas JA Stalpers, Steven E Schild, Dirk Rades

**Affiliations:** 1 Department of Radiation Oncology, University of Lubeck, Germany; 2 Department of Radiation Oncology, Dr. Bernard Verbeeten Institute Tilburg, The Netherlands; 3 Department of Radiation Oncology, Academic Medical Center Amsterdam, The Netherlands; 4 Department of Radiation Oncology, Mayo Clinic Scottsdale, USA; 5 Department of Radiation Oncology, University of Lubeck, Germany and Department of Radiation Oncology, University of Hamburg, Germany

## Abstract

**Background:**

This study investigated potential prognostic factors in patients treated with whole-brain irradiation (WBI) alone for brain metastases from relatively radioresistant tumors such as malignant melanoma, renal cell carcinoma, and colorectal cancer. Additionally, a potential benefit from escalating the radiation dose was investigated.

**Methods:**

Data from 220 patients were retrospectively analyzed for overall survival and local control. Nine potential prognostic factors were evaluated: tumor type, WBI schedule, age, gender, Karnofsky performance score, number of brain metastases, extracerebral metastases, interval from diagnosis of cancer to WBI, and recursive partitioning analysis (RPA) class.

**Results:**

Survival rates at 6 and 12 months were 32% and 19%, respectively. In the multivariate analysis, WBI doses >30 Gy (p = 0.038), KPS ≥70 (p < 0.001), only 1-3 brain metastases (p = 0.007), no extracerebral metastases (p < 0.001), and RPA class 1 (p < 0.001) were associated with improved survival. Local control rates at 6 and 12 months were 37% and 15%, respectively. In the multivariate analyses, KPS ≥70 (p < 0.001), only 1-3 brain metastases (p < 0.001), and RPA class 1 (p < 0.001) were associated with improved local control. In RPA class 3 patients, survival rates at 6 months were 10% (35 of 39 patients) after 10 × 3 Gy and 9% (2 of 23 patients) after greater doses, respectively (p = 0.98).

**Conclusions:**

Improved outcomes were associated with WBI doses >30 Gy, better performance status, fewer brain metastases, lack of extracerebral metastases, and lower RPA class. Patients receiving WBI alone appear to benefit from WBI doses >30 Gy. However, such a benefit is limited to RPA class 1 or 2 patients.

## Background

Malignant melanoma, renal cell carcinoma, and colorectal cancer are considered relatively radioresistant tumors. Little data exist regarding the radiotherapy of brain metastases from such tumors. Brain metastases develop in up to 46% of melanoma patients, 4-11% of renal cell carcinoma patients, and in 0.3-9% of colorectal cancer patients [1-6]. The median survival time of these patients is only a few months [7-9]. Whole-brain irradiation (WBI) alone still is the most commonly administered treatment. The most frequently applied radiation schedule is 10 × 3 Gy in two weeks. In carefully selected patients, more aggressive treatment options including neurosurgery or radiosurgery may be justified to extend survival. However, these more aggressive treatments may be associated with increased risk. Thus, it is important to individualize the treatment approach for each patient taking into account his or her prognosis.

The major objective of the present study was the definition of significant prognostic factors for overall survival and local control following WBI for brain metastases from a relatively radioresistant tumor. Such prognostic factors can help the physician select the appropriate treatment for the individual patient. Additionally, prognostic factors are important for proper stratification in future trials. Additionally, this study investigated the potential benefit from an escalation of the WBI dose beyond 10 × 3 Gy with respect to overall survival and local control.

## Methods

A total of 220 patients who were treated with WBI alone for brain metastases from a relatively radioresistant tumor such as malignant melanoma (N = 69), renal cell carcinoma (N = 74) or colorectal cancer (N = 77) between 1989 and 2008 were included in this retrospective analysis. Further criteria for inclusion were as follows: no prior radiotherapy to the brain, confirmation by computed tomography or magnetic resonance imaging, and administration of dexamethasone (12-32 mg/day) during WBI. The data were obtained from the patients, their general practitioners, treating oncologists, and patient files. The data have been analysed anonymously. The study has been approved by the ethic committee of the University of Lubeck. Patient characteristics are summarized in Table 1. The patients treated in Tilburg received 5 × 4 Gy in one week and those patients treated at the University of Lubeck received 10 × 3 Gy. At the University of Hamburg the radiation schedule varied based on the discretion of the treating physician. Most patients treated in Hamburg received doses beyond 30 Gy, i.e. 15 × 3 Gy in three weeks or 20 × 2 Gy in four weeks.

**Table 1:** Patient characteristics.

	N (%)
**Tumor type**	
Malignant melanoma	69
Renal cell carcinoma	74
Colorectal cancer	77

**WBI schedule**	
5 × 4 Gy	56
10 × 3 Gy	99
15 × 3/20 × 2 Gy	65

**Age**	
≤ 62 years	109
≥ 63 years	111

**Gender**	
Female	78
Male	142

**Karnofsky Performance Score**	
<70	89
≥ 70	131

**Number of metastases**	
1-3	89
≥4	131

**Extracerebral metastases**	
No	68
Yes	152

**Interval from first diagnosis to WBI**	
≤ 24 months	104
≥ 25 months	116

**RPA class**	
1	25
2	105
3	90

WBI was perfomed with 6-10 MV photon beams from a linear accelerator via parallel opposed fields (90° and 270°).

The following potential prognostic factors were evaluated: primary tumor type (malignant melanoma versus renal cell carcinoma versus and colorectal cancer), radiation schedule (5 × 4 Gy in one week versus 10 × 3 Gy in two weeks versus 15 × 3 Gy in three weeks or 20 × 2 Gy in four weeks), age (≤62 versus ≥63 years, median age: 63 years), gender, Karnofsky performance score (KPS <70 versus KPS ≥70), number of brain metastases (1-3 versus ≥4), presence of extracerebral metastases at the time of WBI (no versus yes), interval between first diagnosis of cancer and WBI (≤24 versus ≥25 months), and RPA (RPA 1 versus RPA 2 versus RPA 3).

Gaspar et al. identified three RPA classes based on data from Radiation Therapy Oncology Group (RTOG) brain metastases trials [10]. RPA class 1 patients have a Karnofsky Performance Score (KPS) ≥70, age <65 years, no extracerebral metastases, and a controlled primary tumor. RPA class 2 patients have a KPS ≥70, and at least one unfavorable prognostic factor such as age ≥65 years, extracerebral metastases, or uncontrolled primary tumor. RPA class 3 includes all patients with a KPS <70. The median survival for the RPA classes 1, 2, and 3 were 7.1 months, 4.2 months, and 2.3 months, respectively [10].

Local control was defined as the absence of progressive or recurrent brain metastasis. The diagnosis of progression or recurrence was confirmed by computed tomography (CT) or magnetic resonance imaging (MRI). All patients had CT or MRI scans obtained if neurologic deterioration or other symptoms most likely related to progression of brain metastases occurred. Local recurrence was defined progression of treated lesions or progression elsewhere in the brain. Time to death and to local recurrence was measured from the completion of radiotherapy.

Overall survival and local control rates were calculated using the Kaplan-Meier-method [11]. Differences between the Kaplan-Meier curves were determined with the log-rank test (univariate analysis). The prognostic factors found to be significant (p < 0.05) in the univariate analysis were included in a multivariate analysis, which was performed with the Cox proportional hazards model. The patients were followed until death or for median 8.5 months (range: 6-54 months) in those who were alive at last follow up. If both RPA class and any factor contributing to the RPA class (age, KPS, extracerebral metastases) were significant in the univariate analysis, two multivariate analyses were performed. One multivariate analysis included the RPA class but none of the three other confounding factors and the second analysis included the three other factors but not RPA.

## Results

Median survival time for the entire cohort was 3.5 months. The survival rates at 6 months and 12 months were 32% and 19%, respectively. In the univariate analysis, better overall survival was associated with the WBI schedule (15 × 3 Gy/20 × 2 Gy, p < 0.001), KPS ≥70 (p < 0.001), only 1-3 brain metastases (p < 0.001), absence of extracerebral metastases at the time of WBI (p < 0.001), and RPA class 1 (p < 0.001). The results of the univariate analysis for overall survival are summarized in Table 2. In the multivariate analysis of overall survival, WBI schedule (hazard risk [HR]: 1.23; 95%-confidence interval [CI]: 1.01-1.49; p = 0.038), KPS (HR: 2.06; 95%-CI: 1.49-2.86; p < 0.001), number of brain metastases (HR: 1.18; 95%-CI: 1.05-1.34; p = 0.007), extracerebral metastases (HR: 1.57; 95%-CI: 1.10-2.28; p = 0.012), and RPA class (HR: 2.15; 95%-CI: 1.65-2.82; p < 0.001) maintained significance.

**Table 2:** Univariate analysis of survival.

	At 6 months	At 12 months	
	(%)	(%)	P
**Tumor type**			
Malignant melanoma	32	8	
Renal cell carcinoma	38	30	
Colorectal cancer	26	18	0.08

**WBI schedule**			
5 × 4 Gy	23	8	
10 × 3 Gy	24	14	
15 × 3/20 × 2 Gy	51	37	**<0.001**

**Age**			
≤62 years	38	22	
≥63 years	26	17	0.10

**Gender**			
Female	26	24	
Male	35	18	0.52

**Karnofsky Performance Score**			
<70	7	2	
≥70	49	32	**<0.001**

**Number of metastases**			
1-3	54	38	
≥4	17	6	**<0.001**

**Extracerebral metastases**			
No	57	32	
Yes	20	13	**<0.001**

**Interval from first diagnosis to WBI**			
≤24 months	31	16	
≥25 months	33	21	0.30

**RPA class**			
1	96	61	
2	37	25	
3	8	2	**<0.001**

In the subgroup of the RPA class 3 patients, the survival rates at 6 months were 10% (35 of 39 patients) after 10 × 3 Gy and 9% (2 of 23 patients) after 15 × 3 Gy/20 × 2 Gy, respectively (p = 0.98).

The median time to recurrence for the entire cohort was 4.5 months. The local control rates at 6 months and 12 months were 37% and 15%, respectively. In the univariate analysis, better local control was associated with the WBI schedule (15 × 3 Gy/20 × 2 Gy, p < 0.001), KPS ≥70 (p < 0.001), only 1-3 brain metastases (p < 0.001), and RPA class 1 (p < 0.001). The results of the univariate analysis for local control are summarized in Table 3. In the multivariate analysis of local control, KPS (HR: 1.97; 95%-CI: 1.35-2.86; p < 0.001), number of brain metastases (HR: 1.36; 95%-CI: 1.18-1.57; p < 0.001), and RPA class (HR: 1.88; 95%-CI: 1.41-2.53; p < 0.001) maintained significance, whereas the WBI schedule was not significant (HR: 1.08; 95%-CI: 0.87-1.34; p = 0.46).

**Table 3:** Univariate analysis of local control.

	At 6 months	At 12 months	
	(%)	(%)	P
**Tumor type**			
Malignant melanoma	37	5	
Renal cell carcinoma	41	22	
Colorectal cancer	33	14	0.36

**WBI schedule**			
5 × 4 Gy	42	0	
10 × 3 Gy	20	4	
15 × 3/20 × 2 Gy	53	30	**<0.001**

**Age**			
≤ 62 years	42	13	
≥ 63 years	31	18	0.63

**Gender**			
Female	33	16	
Male	39	14	0.32

**Karnofsky Performance Score**			
< 70	12	0	
≥ 70	50	23	**<0.001**

**Number of metastases**			
1-3	60	28	
≥4	18	0	**<0.001**

**Extracerebral metastases**			
No	45	21	
Yes	32	10	0.10

**Interval from first diagnosis to WBI**			
≤ 24 months	37	10	
≥ 25 months	37	19	0.44

**RPA class**			
1	80	38	
2	42	18	
3	12	0	**<0.001**

Acute toxicity grade ≥2 according to the Common Toxicity Criteria 2.0 [12] occurred in 17/99 (17%) patients who received 30 Gy in 10 fractions, and 12/65 (18%) patients who received greater doses. Neurocognitive deficits most likely related to WBI were observed in 6/99 (6%) patients who received 30 Gy in 10 fractions and 5/65 (8%) patients who received greater doses.

## Discussion

Only limited data exist regarding the treatment of brain metastaes from relatively radioresistant tumors such malignant melanoma, renal cell carcinoma, and colorectal cancer. Farnell et al. presented a retrospective series of 146 patients with brain metastases from colorectal cancer [13]. Thirty-nine patients received surgery plus WBI, 11 patients surgery alone, 79 patients WBI alone, and 17 patients supportive care alone. The median survival times were 42, 45, 16 and 8 weeks, respectively. Ikushima et al. reported median overall survival times of 26 months after fractionated stereotactic radiotherapy, of 19 months after surgery followed by WBRT, and of 4 months after WBRT alone in a retrospective series of 35 patients with brain metastases from renal cell carcinoma and a comparably good performance status [14].

Prognostic factors may aid the clinician select the most appropriate treatment regimen for the individual patient. Several scoring systems exist that allow one to estimate the survival prognosis of patients with brain metastases [10,15]. Brain metastases from different tumor entities vary with respect to their radiobiological behavior. Some entities such as malignant melanoma, renal cell carcinoma and colorectal cancer are considered relatively radioresistant. None of the previously reported scoring systems focused on these particular tumors.

The present study identified prognostic factors associated with survival and local control for patients who received WBI alone for brain metastases from these radioresistant tumor types. On multivariate analyses, overall survival was significantly associated with WBI schedule, KPS, number of brain metastases, extracerebral metastases at the time of WBI, and RPA class. Local control was significantly associated with KPS, number of brain metastases, and RPA class. Regarding the WBI schedule, doses higher than 10 × 3 Gy resulted in better overall survival than standard doses of 10 × 3 Gy and 5 × 4 Gy. Better local control after doses higher than 10 × 3 Gy was observed only in the univariate analysis. In a recently published meta-aanalysis, altered dose-fractionation schedules of WBI did not result in significant differences in median survival and local control [16], which is different from the findings of the present study. This difference can be explained by the fact that the data from the meta-analysis were mostly derived from different, less radioresistant tumor entities. However, the retrospective design of the present study should be considered when interpreting these results. Retrospective studies are always at risk of including hidden biases.

The biological effectiveness of irradiation depends on both the total dose and the dose per fraction. Different radiation schedules can be compared with the Equivalent Dose in 2 Gray Fractions (EQD2), calculated with the equation *EQD2 = D [(d + α/β)/(2 Gy + α/β)]*, as derived from the linear-quadratic model; D = total dose, d = dose per fraction, α = linear (first-order dose-dependent) component of cell killing, β = quadratic (second-order dose dependent) component of cell killing, α/β-ratio = the dose at which both components of cell killing are equal [17]. Assuming a α/β-ratio of 3 Gy for tumor cell kill in relatively radioresistant tumors, the EQD2 of the radiation schedules are 28 Gy_3 _(5 × 4 Gy), 36 Gy_3 _(10 × 3 Gy), 40 Gy_3 _(20 × 2 Gy), and 54 Gy_3 _(15 × 3 Gy), respectively. In the present study, fractionation schedules with a higher EQD2 resulted in better overall survival. However, such a benefit was limited to RPA class 1 or RPA class 2 patients, and did not apply to RPA class 3 patients.

Furthermore, our data suggest that the RPA classification which was based primarily on studies including a large variety of different primary tumors, mostly breast cancer and lung cancer patients, applies well to relatively radioresistant tumors. KPS and extracerebral control are the most important prognostic factors of the RPA classification [10]. The number of brain metastases has been identified as a significant predictor for overall survival in a retrospective series of 1,797 patients, mostly lung cancer and breast cancer patients, treated with different treatment regimens including WBI alone [15]. In that large retrospective analysis, local control was associated with performance status and extracerebral metastases as in the present study. The RPA class has not been investigated in that large retrospective series. However, the RPA class is closely related to the KPS. A potential benefit from WBI doses higher than 30 Gy has been suggested in a previous retrospective series of melanoma patients. Isokangas et al. observed a significantly better median survival time with radiation doses higher than 30 Gy when compared to doses of 30 Gy or less (9.6 versus 4.1 months, p < 0.001) [18]. However, in comparison to our present study, the fractionation schedules of the study by Isokangas et al. were more inhomogeneous with total doses ranging from 9 Gy to 60 Gy and doses per fraction ranging from 2 to 4 Gy. A retrospective study of 46 patients with brain metastases from RCC has been reported by Cannady et al. [8]. Median survival times were 2.7 months for 10 × 3 Gy, 0.4 months for doses <30 Gy and 8.5 months for doses >30 Gy, respectively (P = 0.029). The median overall survival times of the latter two studies and of the present study are summarized in Table 4.

**Table 4:** Results of other studies investigating an escalation of the dose of WBI beyond 30 Gy in patients with brain metastases from a relatively radioresistant tumor.

Reference	Design	N patients	Tumor type	Median survival
**Cannady et al. **[8]	retrospective	46	renal cell carcinoma	30 Gy: 2.7 mos. >30 Gy: 8.5 mos.

**Isokangas et al. **[18]	retrospective	60	malignant melanoma	30 Gy: 4.1 mos. >30 Gy: 9.6 mos.

**Present study**	retrospective	164	various	30 Gy: 3.3 mos. >30 Gy: 9.5 mos

## Conclusions

Improved overall survival was associated with WBI doses higher than 10 × 3 Gy, better performance status, fewer brain metastases, lack of extracerebral metastases, and lower RPA class. Improved local control was associated with better performance status, fewer brain metastases, and lower RPA class. Patients with a relatively favorable prognosis may receive neurosurgery or radiosurgery in addition to WBI as these treatments have been shown to improve survival in select patients. Patients who are treated with WBI alone appear to benefit from an escalation of the WBI dose beyond 10 × 3 Gy. However, such a benefit was limited to RPA class 1 or RPA class 2 patients Further dose-fractionation studies are required to confirm the latter results.

## Competing interests

The authors declare that they have no competing interests.

## Authors' contributions

TM participated in acquisition of data, analysis and interpretation of data, and manuscript writing. CH participated in acquisition of data; JDK participated in acquisition of data. TV participated in acquisition of data. LJAS participated in acquisition of data. SES participated in analysis and interpretation of data, performed the statistical analyses, and participated in manuscript writing. DR was responsible for study conception and design and participated in acquisition of data, analysis and interpretation of data, and manuscript writing. All authors read and approved the final manuscript.

## Pre-publication history

The pre-publication history for this paper can be accessed here:

http://www.biomedcentral.com/1471-2407/10/582/prepub
